# West Nile Virus Infection in Humans and Horses, Cuba

**DOI:** 10.3201/eid1206.051235

**Published:** 2006-06

**Authors:** Maritza Pupo, Maria Guadalupe Guzmán, Roberto Fernández, Alina Llop, Félix Orlando Dickinson, Daniel Pérez, Raúl Cruz, Tayri González, Gonzalo Estévez, Hiram González, Paulino Santos, Gustavo Kourí, Maya Andonova, Robbin Lindsay, Harvey Artsob, Michael Drebot

**Affiliations:** * Tropical Medicine Institute "Pedro Kouri," Havana, Cuba;; † Ministry of Public Health, Havana, Cuba;; ‡ Instituto de Ecología y Sistemática, Havana, Cuba;; § Instituto de Medicina Veterinaria, Havana, Cuba;; ¶ National Microbiology Laboratory, Winnipeg, Manitoba, Canada

**Keywords:** West Nile virus, human and horses, Cuba

## Abstract

A surveillance system to detect West Nile virus (WNV) was established in Cuba in 2002. WNV infection was confirmed by serologic assays in 4 asymptomatic horses and 3 humans with encephalitis in 2003 and 2004. These results are the first reported evidence of WNV activity in Cuba.

West Nile virus was first detected in the Western Hemisphere during an outbreak of encephalitis in New York State in 1999 (*1*). Genetic analyses showed that the virus responsible for the 1999 outbreak was nearly identical to a WNV strain circulating in Israel in 1998 (*2*). Recent outbreaks of WNV disease in the United States and Canada have been accompanied by a high proportion of deaths in birds (*3**,**4*), substantial illness in equines (*4**,**5*), and thousands of cases of severe neurologic disease in humans (*6*). The range of WNV has rapidly expanded across the continental United States and Canada (*7*). WNV infection in humans, equines, and birds in Mexico (*8*), the Caribbean (*9*), and South and Central America (*10**,**11*) shows southward movement of the virus. Because Cuba is close to areas of the United States where WNV is endemic and because of recent evidence that suggests spread of WNV into the Caribbean, surveillance was established to monitor for WNV in Cuba. Beginning in 2002, the Medical Services and Ministry of Agriculture and Veterinarian Services of Cuba established a national surveillance program by using birds, horses, and humans to detect WNV activity. In this report, we summarize the key findings of surveillance activities.

## The Study

The Ministry of Agriculture and Veterinarian Services coordinated the collection of dead birds. A total of 1,217 dead birds were shipped at 4°C to the Tropical Medicine Institute "Pedro Kouri" and identified by ornithology experts. Brain, heart, and kidneys were removed and tested for WNV by using reverse transcription–polymerase chain reaction (RT-PCR) (*12*). Briefly, RNA was extracted by using the QIAmp viral RNA kit (Qiagen, Inc., Valencia, CA, USA). Primers WN212 (5´-TTGTGTTGGCTCTCTTGGCGTTCTT-3´) and WN619c (5´-CAGCCGACAGCACTGGACATTCATA-3´) were used to detect viral RNA. A second RT-PCR with primers WN9483 (5´-CACCTACGCCCTAAACACTTTCACC-3´) and WN9794 (5´-GGAACCTGCTGCCAATCATACCATC-3´) was performed on the same RNA preparation.

Serum specimens from horses in Havana and Havana Province were tested for antibodies to WNV by using a competitive enzyme-linked immunosorbent assay (ELISA) with monoclonal antibodies 3.1112G and 6B6C-1 as described by Blitvich et al. (*13*). We tested 210 serum specimens from horses collected as part of an infectious anemia study. The immunoglobulin M (IgM) test was not performed because horses were never suspected of having WNV and did not have any history of suspected viral encephalitis or other illness or symptoms. An inhibition value >30% was used as the diagnostic criterion to identify flavivirus antibody (Table 1).

**Table 1:** Summary of horse sera tested for evidence of WNV infection by ELISA and PRNT*

Sample no.	% inhibition (ELISA)		90% PRNT titer		
	Anti-WNV	Antiflavivirus	WNV	SLEV	Interpretation
C-7	65.3	65.2	160	20	WNV
C-13	58.9	60.4	320	–	WNV
C-19	63.2	63.3	320	–	WNV
C-20	74.6	62.2	640	–	WNV
C-2	44.4	60.4	–	20	SLEV
C-4	11.6	40.1	–	20	SLEV
C-5	67.5	67.6	–	20	SLEV
C-11	10.5	26.7	–	40	SLEV
C-12	32.2	68.0	–	20	SLEV
C-15	27.4	68.9	–	40	SLEV
C-16	64.6	65.7	–	40	SLEV
C-18	59.1	68.2	–	40	SLEV
C-10	62.7	66.3	80	40	Unidentified flavivirus
C-1	22.3	74.4	–	–	Unidentified flavivirus
C-3	40.5	54.1	–	–	Unidentified flavivirus
C-6	28.5	74.8	–	–	Unidentified flavivirus
C-9	40.9	73.6	–	–	Unidentified flavivirus
C-14	34.8	41.0	–	–	Unidentified flavivirus
C-21	9.9	68.4	–	–	Unidentified flavivirus

*WNV, West Nile virus; ELISA, enzyme-linked immunosorbent assay; PRNT, plaque reduction neutralization test; SLEV, Saint Louis encephalitis virus.

The Cuban Health Ministry and Medical Services division conducted surveillance for encephalitis of unknown origin in patients >30 years of age. Serum and cerebrospinal fluid specimens were shipped at 4°C to the Tropical Medicine Institute "Pedro Kouri." Human sera were screened for WNV IgM and IgG by using commercial IgM and IgG ELISA kits (Focus Technologies, Cypress, CA, USA) according to manufacturer's instructions. Hemagglutination-inhibition (HI) tests were also undertaken with WNV and Saint Louis encephalitis virus (SLEV) antigen (*14*).

Reactive serum samples were further tested by a plaque reduction neutralization test (PRNT) with WNV (NY99, Ontario, Canada, 2001 isolate), SLEV (Parton strain, American Type Culture Collection catalog no. VR-1265), and dengue virus (dengue 2, NG-C strain). PRNT was performed to confirm WNV-specific antibody and was carried out as described previously (*15*) by using a neutral red double-overlay procedure. Horses or human patients were considered seropositive for a particular flavivirus if the 90% PRNT titer for that virus was >4-fold greater than the neutralization titers determined for other viruses used in the assay. Endpoint titrations were defined as the highest dilution of serum that reduced plaque formation by >90%.

Most (58%) of the 1,217 birds tested were resident species of Cuba, primarily chestnut manakins (*Lonchura malaccas*), blue jays (*Cyanocitta cristata*), herring gulls (*Larus argentatus*), yellow-faced grassquits/olive finches (*Tiaris olivacea*), and northern parulas (*Parula americana*). None of the birds were positive for WNV RNA by RT-PCR. We attempted to isolate WNV from most samples, but virus was not cultured. Most dead birds were received in good condition for testing.

Nineteen (9.0%) of the 210 horses had serum specimens with antibodies to flaviviruses (Table 1). Four and 8 animals had WNV- and SLEV-specific antibodies, respectively, in the PRNT. Two horses seropositive for WNV came from Havana City, and 2 others came from Havana Province. Seven serum samples had antibodies to undetermined flaviviruses on the basis of results of neutralization assays, and further virus characterization was not performed. None of the horses, including those that were positive for flavivirus antibodies, showed any signs of illness at the time of serum collection.

Serum specimens from 13 patients with encephalitis were tested for possible WNV infection. Two of these patients (M.M. and K.R.) were identified as part of WNV surveillance in 2003. Both acute-phase and convalescent-phase serum specimens from these patients were positive for flavivirus antibody by IgM and IgG ELISAs (Table 2). Cerebrospinal fluid from patient K.R. was also positive for IgM by WNV ELISA. Acute-phase and convalescent-phase serum samples from M.M. and K.R. were also positive by HI assay and showed seroconversion to WNV. Convalescent-phase serum samples were tested for WNV-specific antibody by PRNT, and the neutralization titers were 320 and 160, respectively. Neutralizing antibodies against SLEV or dengue virus were not detected in the serum specimens of either patient. These persons are the first to have confirmed cases of WNV-associated illness in Cuba. Both patients had histories of febrile illness, muscle weakness, and encephalitis, and both were hospitalized. These persons had jobs that required them to spend large amounts of time outdoors, and they lived in communities in Santi Spiritus and Villa Clara in central Cuba. Another person (O.G., Table 2), who also resided in Santi Spiritus, had a low WNV titer by HI assay but had neutralizing antibodies to WNV, which suggests a past WNV infection. This patient was identified during surveillance in 2004 but may have been exposed to WNV in 2003.

**Table 2:** Summary of human sera tested for evidence of WNV infection by ELISA and PRNT*

Patient code	ELISA result†			90% PRNT titers			
	IgM	IgG	HI titer†	WNV	SLEV	DENV	Interpretation
M.M.	+/+	+/+	40/160	320	–	–	WNV
K.R.	+/+	+/+	20/80	160	–	–	WNV
O.G.	-/-	+/+	20/20	40	–	–	WNV
M.L.	-/-	+/+	20/20	–	640	80	SLEV
A.M.L.	-/-	+/+	20/40	–	320	–	SLEV
R.A.	ND/–	ND/+	1,280	–	80	–	SLEV
P.Y.	ND/–	ND/+	<20	–	40	–	SLEV
M.V.	-/-	+/+	20/40	–	80	–	SLEV
J.A.R.	-/-	+/+	20/40	40	80	80	Unidentified flavivirus
R.C.	-/-	+/+	20/20	–	–	80	DENV
A.P.	-/-	+/+	20/20	–	–	–	Unidentified flavivirus
L.V.	-/-	+/+	20/40	–	–	–	Unidentified flavivirus
D.B.	ND/–	ND/+	ND	–	–	–	Unidentified flavivirus

*WNV, West Nile virus; ELISA, enzyme-linked immunosorbent assay; PRNT, plaque reduction neutralization test; Ig, immunoglobulin; HI, hemagglutination inhibition; SLEV, Saint Louis encephalitis virus; DENV, dengue virus; ND, not done. †Acute-phase/convalescent-phase serum samples.

Serum specimens from the 10 remaining patients were negative for WNV IgM but were positive for flavivirus IgG by ELISA; most of these were also positive by HI assay (Table 2). One person appeared to have been exposed to dengue virus, and 4 had antibodies to unidentified flaviviruses. Five patients had serum with SLEV-specific antibodies. Seroconversions were not demonstrated in any of these persons, so we cannot say whether their illnesses were associated with SLEV or dengue virus infections.

## Conclusions

We report the first evidence of antibodies to WNV in horses and humans in Cuba. The fact that human and horse infections have been detected strongly suggests that a local amplification cycle has been established in Cuba. The mode of entry of the virus into Cuba is unknown.

In North America, avian death from WNV infection has been well documented (*3**–**5*). However, none of the dead birds collected during this study showed evidence of viral infection. Although 1,217 animals were tested, a more intensive dead bird surveillance program may be needed to identify animals that die from WNV infection. Resident bird species in Cuba may be less susceptible to WNV infection, and death rates among birds in the Caribbean may be lower than those observed in Canada and the United States.

This study also provides evidence that suggests WNV and SLEV may co-circulate in Cuba. Further studies are required to confirm these observations and to characterize the transmission cycles involved. Finally, expansion of existing mosquito control programs in Cuba, which currently focus on *Aedes aegypti* and dengue prevention, may be required to respond to this new public health threat.
